# Safety during the monitoring of diabetic patients: trial teaching course on health professionals and diabetics - SEGUDIAB study

**DOI:** 10.1186/1471-2458-11-430

**Published:** 2011-06-05

**Authors:** Juan J Cabré, Marta Ripoll, Josep M Hernández, Josep Basora, Ferran Bejarano, Victoria Arija

**Affiliations:** 1 Atención Primaria Reus-Altebrat. Institut Català de la Salut. Tarragona, Spain; 2 IISPV, Universitat Rovira i Virgili, C/Sant Llorenç 21, 43201 Reus, Spain; 3 Institut de Investigació en Atenció Primària. IDIAP Jordi Gol. Barcelona, Spain

## Abstract

**Background:**

Safety for diabetic patients means providing the most suitable treatment for each type of diabetic in order to improve monitoring and to prevent the adverse effects of drugs and complications arising from the disease. The aim of this study is to analyze the effect of imparting educational interventions to health professionals regarding the safety of patients with Diabetes Mellitus (DM).

**Methods:**

*Design*: A cluster randomized trial with a control group.

*Setting and sample*: The study analyzed ten primary healthcare centres (PHC) covering approximately 150,000 inhabitants. Two groups of 5 PHC were selected on the basis of their geographic location (urban, semi-urban and rural), their socio-economic status and the size of their PHC, The interventions and control groups were assigned at random. The study uses computerized patient records to individually assess subjects aged 45 to 75 diagnosed with type 1 and type 2 DM, who met the inclusion conditions and who had the variables of particular interest to the study.

*Trial*: The educational interventions consisted of a standardized teaching course aimed at doctors and nurses. The course lasted 6 hours and was split into three 2-hour blocks with subsequent monthly refresher courses.

*Measurement*: For the health professionals, the study used the *Diabetes Attitude Scale *(DAS-3) to assess their attitudes and motivation when monitoring diabetes. For the patients, the study assessed factors related to their degree of control over the disease at onset, 6, 12 and 24 months.

*Main variables*: levels of HbA1c.

*Analysis*: The study analyzed the effect of the educational interventions both on the attitudes and motivations of health professionals and on the degree of control over the diabetes in both groups.

**Discussion:**

Imparting educational interventions to health professionals would improve the monitoring of diabetic patients. The most effective model involves imparting the course to both doctors and nurses. However, these models have not been tested on our Spanish population within the framework of primary healthcare.

**Trial registration:**

ClinicalTrials.gov: NCT01087541

## Background

Safety for diabetic patients refers both to the way in which they are handled and to the suitability and pharmacological safety of the treatment they are given. This is not just a financial issue but a broader concept which means providing suitable healthcare that is in line with the latest findings [1].

We have known for some time that getting diabetic patients to perform repetitive self-measurements of capillary glycemia does not improve the way in which they are monitored [2,3]. Therapeutic educational measures are better. Although they seem more complex, their performance is more optimal [4-6].

A broad consensus is lacking on several issues concerning the treatment of diabetic patients. These issues include the systematic use of anti-aggregant treatment [7], the use of statins [8], the use of ACE inhibitors or ARB (even when the patient is not hypertensive) [9], the need to calculate the cardiovascular risk and the value of classifying diabetic patients as high-risk. The answers to all these issues may vary depending on the method used to resolve them.

According to a recent review of medical errors in diabetes, patient follow-up can often include such problems as drug incompatibilities, contraindications, therapeutic inertia, a lack of desirable monitoring and the non-detection or control of other diabetes-associated risk factors. Some estimates state that there is a 63.2% chance that these problems will occur over a year (22% for glycemia monitoring, 58% for lipid monitoring and 10% for inappropriate prescription) [10]. Clinical practice guides have attempted to partially resolve this problem. Each guide sets out the guaranteed minimum care level and recommends preventive measures or screening. Nonetheless, the heterogeneous nature of these guides prevents them from being effectively applied in BHA; that is, they provide highly homogeneous results that conceal any significant differences at either individual or group level [11-13].

Over the last few years we have seen many advances in the treatment and handling of diabetic patients. Some of these treatments use rosiglitazone or pioglitazone; however, their adoption has been limited by the increased risk of heart failure and ischemic coronary events associated with the use of rosiglitazone, and the increased risk of bone fractures in women treated with rosiglitazone or pioglitazone [14,15]. Other studies have tried to develop safer treatments such as insulin analogs [16]. Although the principal obstacle to improving new treatments is often financial, sometimes it can be therapeutic inertia, which is very difficult to resolve [17].

We should point out that in our evidence-based assessment of therapeutic alternatives we have considered the points recommended by the World Health Organization (WHO) regarding essential drugs. These points refer to the efficacy, safety, financial cost and ease of administration of a given drug. Examples of these points can be found in the following list of measures relating to diabetic patient safety: 1) creating consensual diagnostic criteria and defining abnormal baseline glycemia and glucose intolerance as prediabetic conditions; 2) not marketing a glitazone (troglitazone) in Spain because of serious liver problems observed in the United States [18,19]; 3) describing potentially hazardous interactions between antidiabetic drugs or between antidiabetics and other drugs in polymedicated patients; 4) reaffirming the efficacy of programs for weight loss and physical exercise as a means of prevention [20,21]; 5) recommending anti-aggregation treatment for high-risk diabetics; 6) providing insulins and oral antidiabetics with a lower risk of hypoglycemia; 7) improving metabolic monitoring (glycosylated hemoglobin); 8) disseminating screening and education programs regarding complications of Diabetes Mellitus (DM) such as diabetic foot, ECG, Doppler; 9) setting up a non-mydriatic camera program to detect diabetic retinopathy; 10) providing lifelong diabetology training for the family doctor; 11) monitoring certain parameters of the diabetic patient by means of a computerized program for managing clinical histories within the field of primary care; 12) setting up an oral health program in diabetics; 13) carrying out diabetes prevention programs.

Given the somewhat uncertain situation regarding the monitoring and follow-up of diabetic patients, is reasonable to think that whether the monitoring of diabetes patients improves in the medium-term when the doctors and nurses who care for them are provided specific lifelong training which prioritizes their professional approach to these patients [22,23]. Therefore, with the aim of improving the safety of diabetic patients, we would like to know how educational interventions affect the way in which health professionals monitor diabetic patients.

## Methods

### Design and study period

A cluster randomized trial controlled was carried out in primary healthcare centres (PHC). Ten PHC were distributed randomly into two groups: five PHC in the intervention group and five PHC in the control group.

**Figure 1:**
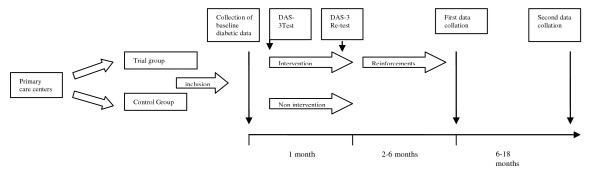
**Study design**.

The study ran from November 2007 to December 2009. The intervention was aimed at health professionals lasted six months. At the start of the intervention period a test was performed on the health professionals' attitudes and motivations towards diabetics; a re-test was performed at the end of this period. During the next 5 months, monthly refresher tests were given. After this, the effect of the intervention given to health professionals working with patients with DM was estimated at baseline to 6, 12 and 24 months after the intervention. During the inclusion period, the baseline data of the diabetic patients were obtained from the 10 PHC (Figure 1).

### Study population

HbA1c was used as the principal variable for calculating the sample size. On the basis of previous studies, reducing HbA1c by 0.25% over 1 year was assumed to be clinically significant. Therefore, on the basis of an alpha risk of 0.05 and a beta risk of 0.10 in a bilateral comparison, 337 subjects were needed for each group. In order to compute the effect of the design, the size of the sample was multiplied by 2 (n = 674), and 5% was added for possible recording errors, and 10% for losses during patient follow-up. The final sample was 775 subjects in each group.

The population was selected in 3 phases:

#### Phase 1: Selection of the PHC

The study was performed in a geographic area covering 32 PHC (primary healthcare centers) of the Catalan Health Institute from Tarragona-Reus, Catalonia, Spain. Ten PHC with different geographic locations (urban, semi-urban and rural) and socio-economic statuses (high-medium and medium-low) were selected, representing approximately 150,000 inhabitants. According to our own data, there were 7,350 subjects in the 10 PHC with type 1 and type 2 DM (DM1, DM2) and aged 45-75. Consequently, we had a sufficiently large database to eliminate subjects who did not comply with the exclusion criteria.

#### Phase 2: Participation of professionals

All health professionals from the 5 PHC of the intervention group were informed about the study and of the possibility of participating in a voluntary manner. If they accepted they had to sign an informed consent form. Joint participation of the doctor and nurse team was the only criteria for selection.

#### Phase 3: Selection of patients with DM

All the diabetic patients that were seen by professionals from the 10 PHC were included in the study. The inclusion criteria were being diagnosed with DM1 and DM2 in the primary-care patient computer records, and being aged 45-75. The exclusion criteria were: not having the study variables, suffering from a serious terminal disease or being monitored exclusively by a specialist (endocrinologist or other).

### Allocation of study groups

To ensure that the intervention group and the control group were comparable, they were paired with another center with similar characteristics in terms of area (urban/semiurban/rural), socio-economic status, cultural status and size of the PHC. Pairs were distributed into 2 groups and then randomly designated as the intervention group and the control group.

### Intervention

The intervention consisted of imparting a series of teaching courses on DM [24] at each of the centers of the intervention group. Each course involved 6 teaching hours split into 3 blocks of 2 hours, in which both doctors and nurses participated. The content of the sessions was standardized and taught by 3 primary care doctors and a clinical pharmacologist, each person imparting a particular section of each session. A primary care doctor presented the project, the other two doctors presented the remaining part of the course and the workshops, and the clinical pharmacologist presented the section on drug safety.

The study was standardized into the following 3 blocks. On the first day the project was presented and the participants were asked to fill in the questionnaire on their attitudes and motivations regarding diabetes, the *Diabetes Attitude Scale *(DAS-3). The most important adverse effects and pharmacological interactions were also explained. On the second day the workshop on therapeutic education was held to explain concepts regarding adult education prioritize activities and hold experience and reflection workshops. On the third day the current pharmacological treatment of diabetes were examined and clinical practice guides for diabetes were reviewed. At the end of the sessions, participants were asked to fill in questionnaire DAS-3 again.

The main aim of the educational sessions was to encourage the participants to reflect on the aspects of communication and learning that emerged during each workshop. These involved awareness sessions in which, professionals did tactile discrimination, verbal and non-verbal communication or trust building exercises.

The pharmacological warnings section reported the safety profile of the drugs used in diabetics (oral antidiabetics and insulin), thus allowing us to identify possible adverse effects and interactions.

In addition to the face-to-face sessions, monthly reminders were issued both in paper and electronic format to all the professionals participating in the intervention group. This material included: 5 information bulletins (pharmacological warnings, criteria for monitoring DM, periodic trials to be performed, dietary and physical exercise advice) and suggestions from the DESG group (*Diabetes Education Study Group*).

A record of each patient's hypoglycemic status was given to the professionals. This included: the patient's sex, the number of years he/she had DM (divided into three groups: less than 5 years, 5 to 10 years and more than 10 years), the type of treatment usually received, and confirmation or not of the presence of hypoglycemia and its possible causes. A record of each patient's admissions to hospital due to diabetes related conditions (including hypoglycemia, non-ketotic hyperosmolar condition, ketoacidosis, amputation, ischemic cardiopathy, diabetic neuropathy, etc) was also given.

Someone was available throughout resolve any queries from the professionals.

During this educational phase various aspects were considered. On the one hand, we looked at the ability of all health members to access training sessions and the current limited recommendations regarding the type of education and the frequency of the sessions they should have. It seems that to have the maximum effect, sessions should follow certain principles of good clinical practice:

- Courses should follow an adult education methodology.

- Education should be provided by a multidisciplinary team with group leadership skills.

- Sessions should be made accessible to the majority of professionals by taking into account the professionals' availability.

- Educational programs should use several techniques to promote active learning, to adapt to the reality of Health-Primary Care, to take into account local differences and to ensure they can be integrated into normal clinical practice.

### Control group

The management of each center was informed about the study. Professionals from these centers were able to continue their normal clinical practice and were able to carry out their own training following their own criteria and using guides or courses not included within the intervention.

### Measurements

Two sets of results are monitored separately: One for the effect of the intervention on the heath professionals and the other on the diabetic patients.

To assess the effect of the intervention on health professionals the DAS-3 questionnaire is used, which was validated in Spain by one of the members of the study group (both in Spanish and Catalan) [25,26]. The DAS-3 is a questionnaire regarding professionals' attitudes and knowledge towards of diabetics. It comprises 33 questions covering various aspects such as the need for special training, the perception of the seriousness of DM, the assessment of strict monitoring, the assessment of the psychosocial impact of DM, and patient autonomy. The questionnaire was filled in by professionals at the start and end of the month-long course.

To follow-up the diabetic patients, several parameters were defined which could be monitored using the patients' clinical histories from their Primary Care computer records. These parameters were monitored at the start of the intervention and at 6, 12 and 24 months after the intervention on health professionals.

The control items monitored are: HbA1c, cardiovascular risk score, ECG, aspirin or other anti-aggregates treatment, blood pressure, LDL-cholesterol, tobacco habit, body mass index.

The optimal systolic and diastolic blood pressure values are less than 130 and 85 mmHg respectively, according to the guide from the Catalan Health Institute.

The optimal LDL-cholesterol values are less than 100 mg/dL according to the American Diabetes Association guide [24].

The control items did not include the number of visits made by diabetics to the doctor's because the reasons for such visits can vary greatly. Consumption of strips for selfcontrol was also not taken into account because this does not correlated with degrees of control or with cardiovascular complications when the follow-up period is short-term.

### Statistical methods

The variables collated for the professional and patient are reported as mean and standard deviations and as percentages.

A chi-squared test, *t *student Fisher's and analysis of variance were used to compare means and proportions. Kolmogorov-Smirnov tests were used to verify the hypothesis regarding the normal distribution of quantitative variables. In the case of non-compliance with test conditions, non-parametric tests were applied.

The results of the DAS-3 test, before and after the educational intervention, were compared between the intervention and control group. The differences between doctors and nurses were also estimated.

The results from variables recorded in the patient were compared in both groups before the educational intervention to ensure their comparability. The results before and after of the educational intervention were then compared for both the intervention and the control group.

The effects of the educational intervention were measured by adjusting with other variables related to the monitoring of DM, such as age, sex, BMI, the socio-economic status of the PHC, and any other characteristic depending on the model used. Consequently, the multivariate statistical methods of multiple linear regression and logistical regression were used. Conditions for applying models were verified mainly by analyzing residuals. During the first phase, a theoretical model including all the variables was made. During the second phase, automatic selection using the backward and forward method was carried out to obtain more reduced models.

In all cases the level of significance was set at p < 0.05.

Version 17.0 of the statistical programs package SPSS/PC for Windows was used to analyze data.

### Ethical Aspects

The study protocol was evaluated and approved by the Ethics Committee of Assistance (CEA) of the Institut d'Investigació en Atenció Primària, Jordi Gol (IDIAP Jordi Gol). Informed consent was obtained from the health professionals in both the trial and control groups.

### Forecast execution dates

- Protocol design: October - November 2007.

- Presentation of the study to health centers: December 2007

- Intervention period: January 2008

- Period for sending reinforcements: February - June 2008

- Initial data collation: July-August 2008

- Subsequent data collations: July-August 2009

- Publication of final results: December 2010

## Discussion

There is currently no broad consensus regarding the treatment and follow-up of diabetic patients. Some researchers have highlighted that a high percentage of health professionals find it difficult to follow up diabetic patients [10]. Although recommendations have attempted to resolve these difficulties, it seems that general monitoring of diabetic patients continues to be defective [11-13]. Furthermore, recent medical advances in treating diabetic patients have often not been translated into recommendations for providing primary care treatment and monitoring [17]. Therefore, it is considered important to provide specific lifelong and structured training to health professionals with the purpose of improving the medium-term monitoring of diabetic patients in primary care centers.

The study was designed to randomly select a limited number of basic health areas concentrated in a geographic area and with similar socio-demographic and cultural characteristics, thus guaranteeing the homogeneity of the patients in each group.

In addition, the socio-demographic and cultural characteristics of our geographic area correspond to mean levels in other basic health areas of industrialized countries, which also means these results can be applied to similar fields.

The training methodology performed in this study consisted of short educational sessions, with periodic reinforcements, which represents a new method when compared with the traditional methods used in different primary care centers. This is because it prioritizes interactivity of all professionals through practical workshops. The method also prioritizes the group knowledge rather than the individual knowledge. Another important point is to use the questionnaire on attitudes and knowledge about diabetes and the analysis within the group to determine the weakest points perceived by professionals so that these can be resolved.

This study has been disseminated electronically because it is a more environmentally friendly form of giving feedback than using paper. New technologies (video, music and photography) were also used to improve communication.

To summarize, the SEGUDIAB program can be considered an useful method for improving the care of diabetic patients because it provides tools and knowledge for modifying the attitudes of health professionals.

## Competing interests

The authors declare that they have no competing interests.

## Authors' contributions

The study's chief researchers JJC and JMH were responsible for identifying the research question, the design of the study, obtaining ethics approval, the acquisition of funding, analysing the data, writing the manuscript and overseeing the study. VA was responsible for the acquisition of funding, analysing the data, writing the manuscript and overseeing the study. MR, JMH, JB, FB to developing the precise content of the study interventions and resources, recruiting participants and study implementation and wrote the paper. All authors were responsible for drafting the manuscript and read and approved the final version.

## Acknowledgements

This study was supported by a grants from the Fundació Avedis Donabedian (Universitat Autònoma de Barcelona), Spain.

## Pre-publication history

The pre-publication history for this paper can be accessed here:

http://www.biomedcentral.com/1471-2458/11/430/prepub
